# Secondary Plant Products Causing Photosensitization in Grazing Herbivores: Their Structure, Activity and Regulation

**DOI:** 10.3390/ijms15011441

**Published:** 2014-01-21

**Authors:** Jane C. Quinn, Allan Kessell, Leslie A. Weston

**Affiliations:** 1 School of Animal and Veterinary Sciences, Graham Centre for Agricultural Innovation, Charles Sturt University, Boorooma Street, Wagga Wagga, NSW 2678, Australia; E-Mails: jquinn@csu.edu.au (J.C.Q.); akessell@csu.edu.au (A.K.); 2 Graham Centre for Agricultural Innovation, Charles Sturt University, Boorooma Street, Wagga Wagga, NSW 2678, Australia

**Keywords:** toxicity, photosensitization, plant, secondary products, biosynthesis, light, UV, dermal tissues, circulatory system

## Abstract

Photosensitivity in animals is defined as a severe dermatitis that results from a heightened reactivity of skin cells and associated dermal tissues upon their exposure to sunlight, following ingestion or contact with UV reactive secondary plant products. Photosensitivity occurs in animal cells as a reaction that is mediated by a light absorbing molecule, specifically in this case a plant-produced metabolite that is heterocyclic or polyphenolic. In sensitive animals, this reaction is most severe in non-pigmented skin which has the least protection from UV or visible light exposure. Photosensitization in a biological system such as the epidermis is an oxidative or other chemical change in a molecule in response to light-induced excitation of endogenous or exogenously-delivered molecules within the tissue. Photo-oxidation can also occur in the plant itself, resulting in the generation of reactive oxygen species, free radical damage and eventual DNA degradation. Similar cellular changes occur in affected herbivores and are associated with an accumulation of photodynamic molecules in the affected dermal tissues or circulatory system of the herbivore. Recent advances in our ability to identify and detect secondary products at trace levels in the plant and surrounding environment, or in organisms that ingest plants, have provided additional evidence for the role of secondary metabolites in photosensitization of grazing herbivores. This review outlines the role of unique secondary products produced by higher plants in the animal photosensitization process, describes their chemistry and localization in the plant as well as impacts of the environment upon their production, discusses their direct and indirect effects on associated animal systems and presents several examples of well-characterized plant photosensitization in animal systems.

## 1. Introduction: Bioactive Secondary Products Produced by Plants—Their Role in Plant Defense

Plants and their associated microflora produce a vast assortment of natural products in their respective metabolomes. Many natural products do not appear to participate directly in plant growth and development, and in some cases their roles are not well understood. Traditionally, these compounds are referred to as secondary plant products (SPPs) and certain families of plants exhibit great metabolic specificity in the SPPs they produce. Primary metabolites are generally found in all plants and are thought to play important roles in essential housekeeping functions [1–3], whereas SPPs play important roles in chemical signaling and defense against grazing herbivores, insects, pests and even other plants [2,4,5]. Given that plants are sessile organisms, it is now well understood that SPPs directly impact plant communication and protection by influencing interactions with other organisms in their environment [1,6,7].

Advances in our ability to identify and detect secondary products at trace levels in the plant and surrounding environment, or in organisms that ingest plants, have provided additional evidence for the role of secondary metabolites in defense or chemical signaling in plant interactions with herbivores, microbiota, nematodes or insects [1,3,7]. In recent years, the once controversial role of SPPs in plant interactions, including allelopathy, has been well-documented; there are now many examples of allelopathic crops and weeds negatively influencing the successful establishment of other plants via chemicals exuded or released into the environment [3,5,8–10]. A smaller subset of SPPs is associated with interactions between plants and their herbivores, which include grazing animals as well as insects and arthropods. Some of these compounds are critically important in the attraction and deterrence of herbivores, one form of chemical communication or signaling [4,7,11].

## 2. Photosensitizing Plant Compounds Affect Grazing Herbivores

The production of SPPs that cause reduced herbivory through repellency, toxicity or irritation is one strategy that has most likely evolved as a selective adaptation by sessile plants for defense against herbivores that selectively feed on them. In grazing livestock, the occurrence of continued irritation or toxicity can result in conditioned learning or avoidance of selected plants, as demonstrated by choice preference tests or trials [12–14]. Grazing animals can use both behavioral and physiological adaptations to reduce the risk of irritation and potential poisoning over time [14]. Despite the use of avoidance and detoxification strategies, both noxious weeds and managed crops ultimately cause millions of dollars in losses in the global livestock industries due to their ability to cause photosensitization and toxicity in both humans and grazing herbivores [15].

Photosensitivity in animals is defined as a severe dermatitis that results from a heightened reactivity of skin cells and associated dermal tissues upon their exposure to sunlight, following ingestion or contact with plant pigments or secondary products that are UV or light reactive [15,16]. Photosensitivity occurs in animal cells as a reaction that is mediated by a light absorbing molecule, in this case the plant—produced secondary products; however, microbial or fungal metabolites can also generate similar responses [15,17,18]. This reaction is most severe in non-pigmented skin where exposure to light is least protected [19]. Photosensitization is not equivalent to ‘sunburn’ although its appearance can be superficially very similar. Sunburn is caused by extended exposure of normal skin to damaging UV rays, while photosensitivity manifests as a rapid reaction of cells in the skin to both visible and UV irradiation through stimulation in the associated light spectrum. Both UV and visible irradiation can cause photoexcitation of light reactive molecules in plants resulting in photosensitivity. In contrast, sunburn typically results from excessive UV exposure, while premature aging is particularly exacerbated by UVA irradiation, due to its ability to penetrate the skin more deeply thereby causing cumulative photodamage to skin [20].

While the precise mechanism of the photosensitization reaction is not well characterized in plants, it is understood to be a light- enhanced oxidation resulting in light-mediated reactions within the plant and also potentially in skin cells of affected grazing herbivores, in response to light frequencies causing direct damage to the proliferative layers of the dermis [15,19]. Photo-oxidation in the plant is associated with production of molecules that result in the generation of reactive oxygen species; some of these may result in free radical damage and some form singlet oxygen as a result of the photodynamic process, leading to eventual DNA degradation in plant cells [18,20]. These cellular changes are also noted in herbivores that have been impacted by photosensitization, and in this case are associated with a similar accumulation of plant-produced photodynamic molecules in the affected tissues or circulatory system of the herbivore. Other photodynamic molecules form photoadducts that interact directly with DNA resulting in photosensitization in grazing herbivores; in this case oxygen is not involved in light-activated photosensitization [18]. Photodynamic molecules generally accumulate in livestock as a result of grazing and are then activated in the skin in the presence of sunlight, after circulation or by direct contact, eventually resulting in DNA degradation upon excitation with UV and/or visible light [20,21]. However, indirect methods of photosensitization can also occur and are generally associated with compromised liver function in grazing livestock [18].

This review on photosensitization aims to: (1) outline the role of unique SPPs produced by higher plants in the animal photosensitization process; (2) describe their chemistry and localization in the plant as well as impacts of the environment upon their production; (3) discuss their direct and indirect effects on associated animal systems; and (4) present several examples of well-characterized plant photosensitization in animal systems. In reviewing the literature on this subject, we have found that there are also many plants that produce SPPs that cause skin irritation but not necessarily photosensitization. In other cases, photosensitization has only been characterized and related secondary products associated with skin damage remain unidentified, indicating potential opportunity for additional research on this subject from the standpoint of the plant and its associated chemistry. However, in this review we will evaluate several well-documented cases as examples of photosensitization and will consider their impacts on grazing herbivores. We also present recent research approaches used to investigate photosensitization associated with a recently introduced summer annual pasture legume in Australia, biserrula (*Biserrula pelecinus* L.) (Papilionoideae) in which the active constituent causing photosensitization is unknown.

## 3. The Role of Light Reactive Molecules in the Photosensitization Process

Plants capture light energy through light-harvesting systems which use plant pigments, including the chlorophylls and carotenoids, for energy capture. Photosynthetic electron transport is then driven in the thylakoid membranes of plant chloroplasts, where ATP and NADPH produced by electron transport in the chloroplast are utilized in subsequent photosynthetic processes including the Calvin cycle [21]. In a photosynthesizing plant, some molecules reach an excited state by absorption of light energy (photons) to become reactive. Chlorophyll is one such ubiquitous light reactive molecule that works effectively to transfer energy in the plant to other molecules, resulting in photosynthesis and eventual plant growth [18]. Under certain environmental and physiological conditions, photosynthetic electron flow or efficiency can be enhanced in the plant [22]. When high light energy conditions occur or plant growth conditions result in maximal production of reactive molecules, photosensitization in grazing herbivores can occur [19]. However, to understand how photosensitization occurs, one needs to understand the process of energy transfer in the plant.

**Figure 1.**
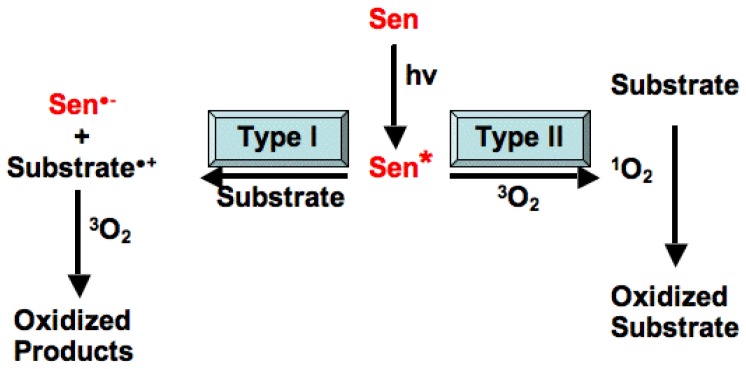
Type I and II chemical photosensitization. Type I: the activated sensitizer reacts with the acceptor, in a one-electron transfer reaction, to produce a radical ion in both the sensitizer and eventually in the acceptor. The acceptor (substrate) donates an electron to the sensitizer (Sen), resulting in an acceptor radical cation (Substrate+) and a sensitizer radical anion (Sen^•−^), but the opposite may also occur, depending on the redox potential of the pair of molecules. In the presence of oxygen, both of these radicals can proceed to produce oxygenated products. Type II reactions: activated sensitizer transfers its excess energy to ground state molecular oxygen, producing excited state singlet oxygen and regenerating the ground-state sensitizer. Singlet oxygen then reacts with the acceptor to produce oxidized metabolites. Reprinted with permission of the author and publisher [24].

In the plant, as well as in any exogenous chemical reactions outside of the plant involving photosensitization, the light-absorbing molecule, in this case chlorophyll or another reactive molecule, is referred to as the photosensitizer and the molecule that is altered by the photosensitizer as the substrate, or acceptor. A chromophore is the part of the molecule that absorbs light of a particular wavelength range. All photosensitizers contain a chromophore whereby absorption of light leads to photochemical changes within the molecule that result in changes in other associated molecules (such as a biological substrate). If the photoactivated photosensitizer produces changes that involve oxygen, the process is referred to as photodynamic. Some photosensitizers, including chlorophyll, are endogenous in the plant, and are physically protected from photodynamic change by cellular machinery and localization in the chloroplast [21,23]. Others are converted by metabolism in the acceptor into a fully functional photosensitizer [24]. It is important to note that chemically there are two main types of photosensitization caused by reactive SPPs, Type I and Type II (Figure 1) [18,24]. In Type I, the activated sensitizer reacts with the acceptor, in a one-electron transfer reaction, to produce a radical ion in both the sensitizer and in the acceptor. Generally, the acceptor (substrate) donates an electron to the sensitizer, resulting in an acceptor radical cation (acceptor+), and a sensitizer radical anion (sensitizer^−^). However, the opposite may also occur, depending on the redox potential of the pair of molecules. In the presence of oxygen, both of these radicals can proceed to produce oxygenated products, leading to the loss of the sensitizer molecule since it is oxidized. Another potential reaction, involving the direct transfer of the extra electron of sensitizer^−^ to oxygen, may also occur to produce a superoxide radical (O_2_^−^), thus regenerating the original sensitizer. In Type II reactions, an activated sensitizer transfers its excess energy to ground state molecular oxygen, producing excited state singlet oxygen and regenerating the ground-state sensitizer. Singlet oxygen then reacts with the acceptor to produce oxidized metabolites. In the case of Type II reactions, the sensitizer is not consumed. Differentiating between type I and type II reactions is not an easy task, especially in living systems [25]. Researchers can generally try to differentiate between the two reaction types by study of the reaction using additives or quenchers which can reduce or extend the excited life of singlet oxygen [24].

### 3.1. Production of Photosensitizers in Higher Plants

In higher plants, the production of singlet oxygen is associated with energy transfer from photoexcited compounds [23] (Figure 1). Singlet oxygen is produced constitutively in plant leaves through chlorophylls that act as photosensitizers [26,27]. These photosensitizing chromophores, as described above, include pigments, certain secondary products and some phytotoxins produced by plant pathogens and microbes. SPPs that undergo photoexcitation are of diverse chemical origin and include the quinones, furanocoumarins, polyacetylenes, thiophenes, benzofurans and chromenes, all of which are able to generate reactive oxygen species upon photoexcitation [23]. These compounds have one key feature in common: they undergo type II photochemistry upon absorption of light energy and thus generate singlet oxygen. This is mainly due to the presence of polycyclic aromatic rings or unsaturated double bonds within their chemical structure. These structural features are the chromophores that absorb radiant energy and trigger the electronic rearrangements within the compounds that lead to the formation of reactive oxygen species (ROS), primarily singlet oxygen, but also superoxide, hydrogen peroxide, hydroxyl radical, and other species [28,29]. Simply put, the absorbed energy excites an outer shell electron to a singlet state of these secondary plant products; this excited state undergoes a process known as intersystem crossing to produce the triplet state of the SPP (Sen* in Figure 1). Normal ground-state molecular oxygen is a triplet, and since two triplets react readily, the energy of the triplet Sen* is transferred to (quenched by) triplet oxygen, regenerating the ground-state Sen and exciting ground-state oxygen to singlet oxygen. Triplet states can also be quenched to produce superoxides, and both are chemically reactive in plant and animal systems [26,27]. One commonly used quencher to study these chemical reactions is azide; azide inhibition of photosensitization reactions can provide evidence in support of mechanisms using singlet oxygen as well as hydroxyl radicals [24].

**Figure 2.**
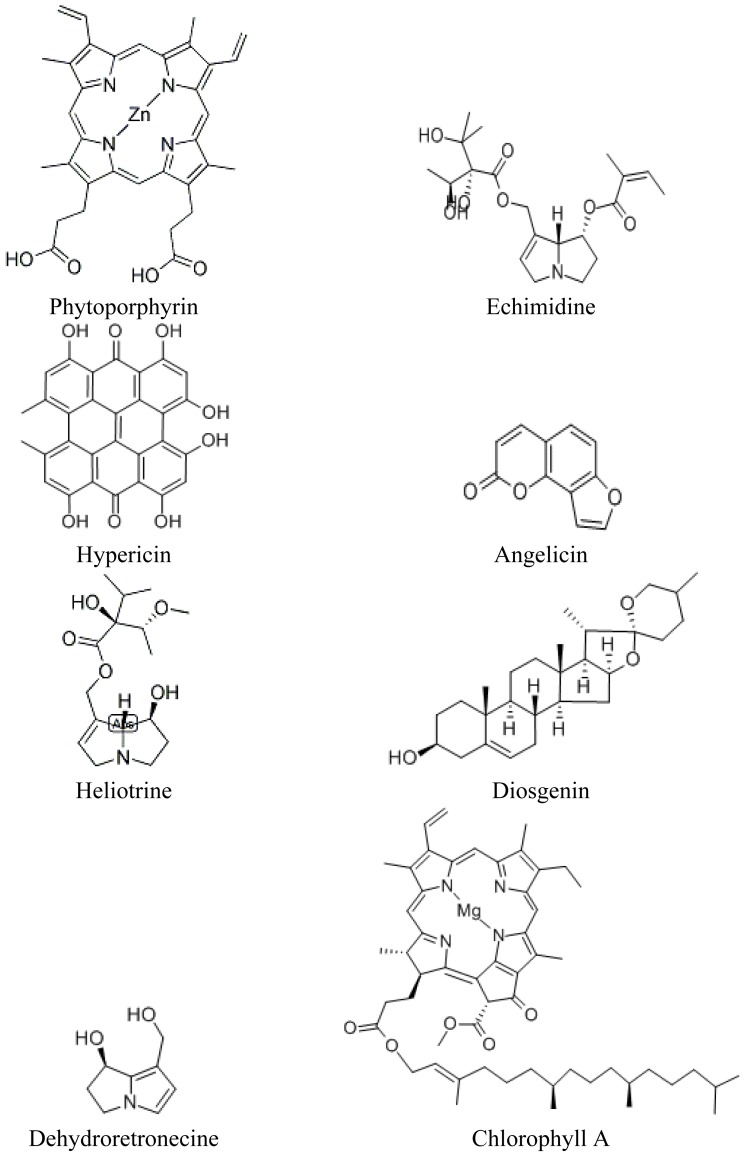
Selected chemical structures of SPPs causing photosensitization in grazing livestock, clockwise from upper left to right. Phytoporphyrin is a microbially produced breakdown product of chlorophyll. Echimidine is produced by *Echium plantagineum*. Angelicin is produced by *Heracleum mantegazzianum*. Diosgenin is produced by *Panicum virgatum*. Chlorophyll A is produced by all green plants. Dehydroretronecine is produced by *Senecio* spp. Heliotrine is produced by *Heliotrope* spp. Hypericin is produced by *Hyperacum perforatum*.

Chlorophylls, the plant pigments responsible for photosynthesis, also act similarly to dyes or other light absorbing compounds in plants and are the source of singlet oxygen and superoxides under certain environmental conditions. Chemically, the ability to form singlet oxygen is fairly widespread throughout the plant kingdom and although many photoreactive plant products have been identified and studied, there are undoubtedly many others that remain as yet unidentified. The structural diversity of these products, their production by varied biosynthetic pathways, and their diverse biogenetic origin in higher plants suggests that these compounds have likely evolved separately and independently in different plants, through adaptive evolutionary processes, as formation of reactive oxygen species may be an important plant defense strategy against herbivores, pathogens and other pests (Figure 2) [4,30].

Interestingly, plants also possess a diverse set of compounds that are capable of protecting themselves against numerous photosensitization reactions that cause damage to the plant’s own membranes and cell structures; these include ubiquitous plant-produced compounds such as the flavonoids, many of which exhibit antioxidant activity [5]. One example is the flavonoid quercetin, an effective quencher of singlet oxygen formation. In the chlorophyll antennae of the plant, carotenoid pigments can also prevent singlet oxygen formation by physically quenching excited chlorophyll molecules and thus preventing the formation of singlet oxygen [31]. After quenching in the plant, excited carotenoids are capable of dissipating excessive energy by release of heat. Other quenching compounds are also present in plant membranes and serve key roles in chemical quenching to prevent overproduction of singlet oxygen. These include the carotenoids, tocopherols, unsaturated fatty acids and also certain proteins. Water-soluble plant quenchers include vitamin B6, ascorbate and various flavonoids [5,31]. The consumption or application of plants or plant extracts containing many of these products has been readily promoted by the pharmaceutical and cosmetic industries in an attempt to protect sensitive skin cells against premature ageing or exposure to harmful UV irradiation.

### 3.2. Regulation and Expression of Photosensitizers in Higher Plants

Reactive oxygen species are produced in all organisms, including plants, in response to environmental stressors and their own metabolism. When plants are grown in or exposed to high light intensity, temperature extremes, or pollutants, reactive oxygen species production has been shown to increase [15,26,27]. In some cases, more reactive species are generated than can be effectively scavenged by various mechanisms for quenching or detoxification in the plant. Plants are also subject to oxidative stress when oxygen is generated in high quantities during photosynthesis [29]. However, under normal growth conditions, the risk of photo-oxidative damage from chlorophyll and its intermediates is relatively low in grazing animals [15]. The production of light-absorbing SPPs in higher plants is generally maximized by exposure to increasing levels of UVA and UVB irradiation, resulting in an enhanced ability to cause photosensitization [32].

The consumption of green plant material rather than dried hay or fodder is also associated with greater levels of photosensitivity in grazing animals. This may be due to the increased prevalence of chlorophylls and protochlorophyllide molecules which are associated with photosensitization in green plant tissues. In addition, concentration of certain SPPs, the heterocyclic molecules causing photosensitivity, is generally highest in green plant material. Active growth of the plant to generate increased biomass is usually associated with high UV exposure and also optimal temperatures and moisture availability in the field. In some instances, dried plant tissues are also the source of secondary plant products causing photosensitivity. In the western USA, seasonal grazing of cattle consuming the dead leaves of *Cooperia pedunculata* Herb. (Liliaceae) a member of the Amaryllis family, resulted in photosensitization [33]. Sometimes a combination of plant materials and SPPs create enhanced photosensitivity in grazing herbivores. In another example from the western USA, photosensitivity in grazing sheep occurred after consumption of horsebrush (*Tetradymia* spp.) (*Helianthus* family), and was increased when sheep concurrently grazed sage (*Artemisia* spp.) (Asteraceae). Horsebrush grows in rangelands with big (*A. tridentata*) and black sage (*A. nigra*); it has been observed that co-consumption has a synergistic effect upon photosensitization [19]. In other cases, it has been reported that cutting and drying plant materials for cured hay or ensilage results in reduced toxicity or photosensitivity in animals ingesting the plant. Many of these recommendations are listed in specific outreach extension reports or documents, based on experiential feeding and handling of animals. However, measurements of SPPs causing photosensitization from dried versus green plant tissues has not generally been performed. Additional chemical characterization of the photosensitizing agents in various plant parts and in processed versus green plant tissues would provide useful information regarding how to ameliorate the problems associated with photosensitization in grazing animals as well.

To date, we have limited information regarding the impact of stress or other biotic and abiotic factors on the plant and its production of photosensitizing agents. Additional research is needed to determine impact of these factors on biosynthetic pathways of photosensitizing compounds and also the potential impacts of global warming or climate change on plant growth, SPP production and formation of photosensitizing compounds in plant tissues. We currently hypothesize, as many others have, that under conditions leading to warmer temperatures, increased UV irradiation and drought stress in green plants, higher concentrations of SPPs could be produced [5,34], which would subsequently lead to greater incidence of photosensitization and plant toxicity in grazing livestock. This is an area that will be critical to address in the near future, as changing climatic conditions have already led to changes in agricultural production and productivity across Australia as well as other agricultural regions. Without having specific datasets yet compiled for additional investigation, current evidence suggests that increasing incidence of plant toxicity and photosensitization is occurring in southern Australia, from anecdotal reports of afflicted livestock in the region as well as consultations regarding affected herbivores.

## 4. Direct and Indirect Effects of Plant-Derived Light-Reactive Molecules in Animal Systems

### 4.1. Photosensitization in Animals

The SPPs responsible for photosensitization trigger complex reactions in sensitive animal skin cells [18,21]. Animal skin typically consists of three major cell layers: the epidermis, dermis and hypodermis [35]. The epidermis is the outermost layer and is the primary protective barrier of the skin and underlying tissues, consisting of a number of cellular layers, some of which are mitotically active. The outer-most layer of epidermis, the stratum corneum (SC), consists of dead keratinized cells that are constantly abraded from the skins surface and replenished by mitotically active cells of the underlying stratum basale (SB). The constant turnover of SB cells allows the SC of the skin to remain intact over time [35]. Disruption of the epidermis, and particularly the SC, diminishes the efficacy of skin as a barrier to exogenous substances or damaging UV irradiation. The deeper layers of integument, the dermis and hypodermis, offer limited or no protection from penetrating substances or light. Therefore, intact epidermal layers are critical to preserve the skin’s protective function. Darker skin containing melanin or pigment also serves as protection to prevent additional photosensitization [19,24]. The presence of light reactive compounds produced by higher plants can result in considerable damage to sensitive dermal tissues, causing irritation, tissue abrasion, secondary skin infection and even death due to associated trauma [35]. In addition, the amino acids in animal skin, including tryptophan, tyrosine and histidine, are particularly susceptible to light- mediated oxidation and eventually invoke significant inflammatory responses in affected tissues and blood vessels that can lead to animal tissue necrosis [15,19].

**Figure 3.**
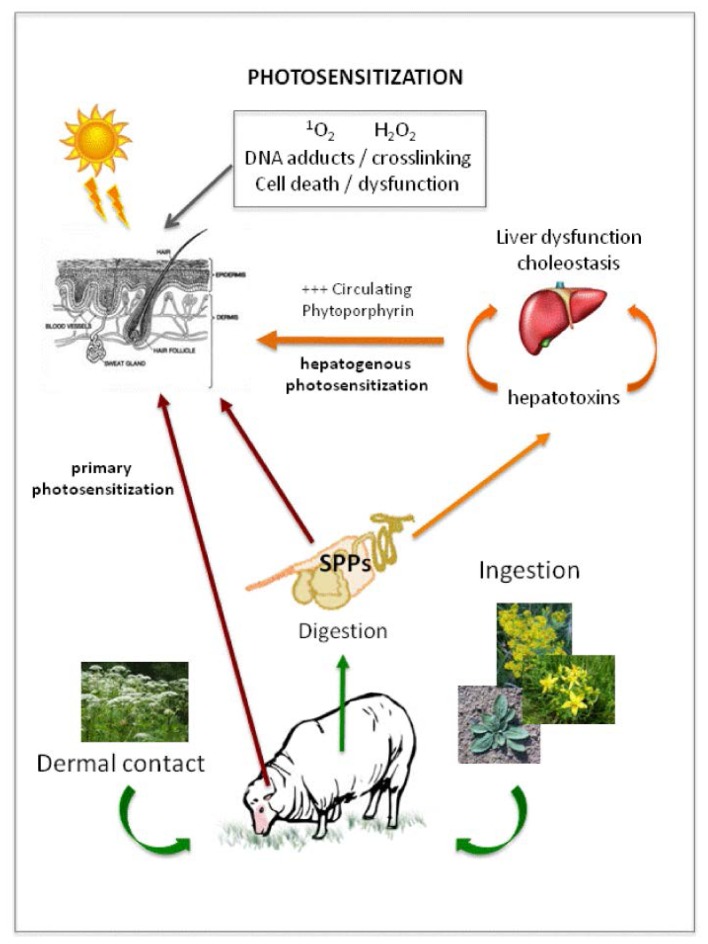
Modes of delivery to the skin of common photosensitizing compounds affecting domestic livestock in primary or hepatogenic photosensitization.

Photosensitivity in grazing herbivores is defined as either primary or secondary photosensitization by veterinary pathologists [15,19]. For the purposes of this review, we will use this nomenclature when referring to types of photosensitization in grazing herbivores. Primary photosensitization occurs when phototoxic plant-produced compounds or their metabolites become bioavailable within the animal after ingestion, or become localized in the cellular layers of the skin (Figure 3). This may be due to direct contact with the plant or from ingestion and dissemination in the herbivore to dermal tissues via absorption from the gut and dissipation in the circulatory system [15]. Primary photosensitization can also develop due to deposition of the products of abnormal porphyrin metabolism in the skin of grazing herbivores. This can arise due to disease resulting from gene mutations in certain mammalian systems that create deficiencies of enzymes involved in the production of hemoglobin, and will not be considered in further detail in this review [15]. Secondary hepatogenic (also referred to as Type 3) photosensitization results from the accumulation of the photodynamic compound phytoporphyrin through the circulatory system of the herbivore. Phytoporphyrin is a microbially-produced metabolite of chlorophyll and is normally cleared from the herbivore’s circulatory system by hepatic metabolism, however, secondary photosensitization may occur as a result of either acute or chronic liver damage in the affected animal when porphyrin and derivatives are not cleared by the damaged liver (Figure 3) [15,36,37].

Primary photosensitivity occurs less commonly in grazing herbivores than hepatogenic photosensitivity [15,19]. Well-characterized examples of polycyclic compounds causing primary photosensitization include the furanocoumarins typically deposited directly on animal skin by contact with plants in the *Apiaceae*, and hypericin (delivered to the skin systemically after ingestion of *Hypericum spp*. (Hypericaceae) containing these compounds) (Figure 2). Although primary photosensitization, by definition, does not result from hepatic failure, a number of primary photosensitizing compounds are also known to cause liver damage, making the distinction between primary and secondary photosensitization less clear in practice [37,38]. Secondary photosensitizers are those that induce liver abnormalities; some can prevent the flow of bile into the duodenum (cholestasis), and there are numerous compounds that are currently reported to damage the liver resulting in secondary photosensitization. The acute and rapid nature of onset of primary photosensitization suggests that compounds associated with this type of photosensitivity are absorbed directly from the gut of the grazing herbivore and then rapidly deposited in animal’s skin, allowing little time for biochemical alteration or significant cholestasis to occur [15,19].

### 4.2. Delivery of Light Reactive Compounds to Animal Systems Resulting in Photosensitization

There are two routes by which photosensitizing natural plant products can enter the skin—either through direct contact, or by systemic transport via the bloodstream (Figure 3) [18,21]. These agents may cause damage to skin cells directly via cytotoxic mechanisms (also referred to as photocytotoxicity), or much less commonly by the induction of an immune system (photoallergic) response. Photocytotoxic photosensitization can show highly variable onset. It can occur within minutes of exposure to the toxic substance by direct contact, within hours (via contact or ingestion) of deposition of the primary photosensitizing agent, or days after exposure due to activation of secondary photosensitizers (following liver damage and deposition of phytoporphyrin into skin). Photoallergic photosensitization may also take days to manifest clinically with either direct or systemic deposition, and is not well characterized in grazing herbivores.

There are two distinct delivery mechanisms by which phototoxic substances can be transported within the dermal layers of affected herbivores following contact exposure to these products. In the first, more polar substances can pass through the skin’s epidermal barrier by active transport processes; in the second, less polar substances pass across dermal layers by passive diffusion [35]. Herbivore skin does possess a number of innate mechanisms to prevent the passage of toxic substances to the sensitive subcutaneous layers. The epidermis provides a relatively high level of resistance to passive transport of bioactive compounds as the keratinized nature of SC cells represents a significant barrier to lipophilic compounds which will preferentially remain within the SC, and also offers resistance to diffusion of polar molecules from traversing into the sub-epidermal layers. The presence of cutaneous lipids also acts as a major barrier to dermal penetration of toxic substances; extraction or breakdown of epidermal lipids has been shown to increase the skin’s permeability to exogenous substances [39]. As such, the matrix of lipids present in skin are both age- and species-dependent; differences in the subcellular properties of the epidermal layers can result in differences in absorptive capacity among grazing herbivores, particularly between different age groups within the same species [39].

Phototoxic substances can also be delivered to the skin via the circulatory system, with compounds present in the systemic circulation reaching the cells of the dermal layers via both passive and active transport mechanisms from the bloodstream. This is a major route of delivery for photocytotoxic compounds in domestic livestock; many of the common cases of photosensitization that are observed are caused by ingestion of compounds resulting in either direct transfer of bioactive molecules to the dermis or subsequent accumulation of secondary products in the dermal layers in response to tissue damage in the liver (Figure 3) [15].

### 4.3. Factors Influencing Sensitivity of Animals to Light Reactive Compounds Causing Photosensitization

**Figure 4.**
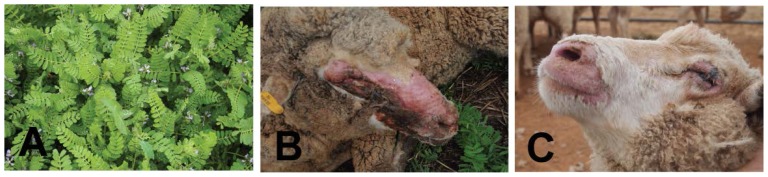
Photosensitization in domestic livestock caused by *Biserrula pelecinus* L. and *Tribulus terrestris* L. (**A**) *Biserrula pelecinus*, a pasture legume crop causing photosensitization in grazing herbivores; (**B**) Severe photosensitization resulting from ingestion of saponins in Australian sheep grazing *Tribulus terrestris*, also known as caltrop. Note severe impact on eyes and face with severe dermal lesions and tissue swelling and loss of fleece on face and muzzle. Eyes are swollen and produce significant exudates; (**C**) Photosensitization in Australian sheep grazing *Biserrula pelecinus* “Mauro”. Lesions are less severe as grazing occurred for only 72 h. Note similar loss of fleece around the muzzle and swelling around the eyes with crusty exudate. This example also experienced corneal ulceration.

The sensitivity of grazing herbivores to dermal toxins or photosensitizing compounds is governed by a number of factors. These include species, breed, skin pigmentation, fur or hide thickness, age, health status of the animal and localized environmental conditions such as temperature, humidity and rainfall [15]. Hair and skin pigment protect the animal from harmful effects of UV exposure, blocking passage of light to the cells of the skin and thus preventing damage to the delicate cellular layers beneath. Therefore, in domestic livestock, photosensitization is most often observed in the areas of skin that remain hairless, such as the skin around the eyes, ears, face, muzzle, mammary gland, tail and the area directly adjacent to the hoof wall (Figure 4) [15,40]. Animals with light-colored or thin coat or fleece covering will also be more affected than their pigmented or thick-coated counterparts [41,42]. Beneath the coat, the cellular characteristics of the epidermal layers can also vary between species [43], and in general, young, sick, non-pigmented or hairless animals are more prone to dermal photosensitization than those that are mature, healthy, pigmented, or hairy. This is due to changes in the ability of the SB to replenish cells of the SC and/or the inherent ability of the skin to be protected from damaging UV irradiation.

## 5. Case Studies Documenting Photosensitization in Grazing Herbivores Due to Exposure to Light Reactive Plant-Produced Compounds

Relatively few SPPs have been clearly identified as causal agents of primary photosensitization due to the complex nature of this pathology and also the difficulty of confirmation of specific mode of action of these compounds and their bioactive metabolites in animal tissues. A smaller number of compounds are confirmed to cause both primary and secondary (hepatogenic) photosensitivity in grazing herbivores, while others are known causal agents of secondary photosensitization. With advances in detection of metabolic compounds using both gas chromatography (GC) and liquid chromatography (LC) coupled to mass spectrometry (MS), particularly quantitative time of flight instruments (QToF), we now have the capacity to identify these bioactive photosensitizing compounds in serum, urine, and skin of grazing herbivores, as well as in the plants that produce them [5,7]. We anticipate a larger number of bioactive photosensitizers will be structurally identified in the not too distant future. Selected examples of plants associated with photosensitization in grazing herbivores, and the causal compounds will now be considered.

### 5.1. Primary Contact Photosensitization and Giant Hogweed (*Heracleum mantegazzianum*) Sommier and Levier

Giant hogweed (*Heracleum mantegazzianum*), a member of the *Apiaceae* family, causes significant skin burns upon contact and subsequent exposure to sunlight. This painful condition is known as phototoxic dermatitis [44–46]. Hogweed grows rapidly and can reach up to 5 m in height; it is found commonly in Europe, North America and its native South-West Asia. Its large leaves are serrated, with dark purple spotting on a stem that is covered in fine bristles. White flowers are contained in characteristic umbels of up to 80cm in diameter during its flowering phase [47]. This large perennial presents a significant human and animal health issue as direct contact with the photosensitizing compounds causes severe burn-like lesions on exposed skin, sometimes leading to amputation or loss of use. Lesions are caused by exposure to both live and cut or harvested plant material [44,48].

The sap of *Heracleum mantegazzianum* contains photosensitizers that cause contact phototoxic dermatitis: a family of photocytotoxic compounds called furanocoumarins [47,49]. The highest levels of furanocoumarins, including angelicin, are found in hogweed leaves but are also present in its stems and inflorescences [50] (Figure 2). Furanocoumarins confer an ecological advantage to the plant, as they possess both insecticidal and antimicrobial properties, thus protecting the plant from pathogen and insect attack [11,51,52]. In mammalian cells, exposure to furanocoumarins causes both carcinogenic and mutagenic changes by virtue of their unusual ability to intercalate directly into host DNA. After integration into the host DNA, exposure to light energy can result in further collation of these bioactive tricyclic molecules which then undergo additional reactions with pyrimidine bases on the complementary DNA strand, giving rise to inter-strand crosslinking [53–55]. Cross-linking within the DNA strands induces apoptosis and inhibits further cell proliferation. Furanocoumarins with mono- or bi-adduct integration ability include angelicin, 5 and 8-methoxysporalen and psoralen [56–59], all of which are present in giant hogweed [47]. The significant skin lesions resulting from dermal contact with the sap of giant hogweed in the presence of sunlight occur due to loss of cell integrity in the dermis, induced by phytogenotoxic DNA damage. Although phototoxic dermatitis caused by giant hogweed is not common in herbivores with a hair coat [60–63], the mechanism of photosensitization is well understood in humans and grazing herbivores.

#### Primary Ingestion Photosensitization and St. John’s Wort (*Hypericum perforatum* L.)

St. John’s Wort (*Hypericum perforatum*) is a perennial weed commonly established in poor and sandy soils of temperate climates. Ingestion of St. John’s Wort by livestock readily causes primary photosensitization with incidences of affected stock being reported across Europe, America and Australasia, including Australia [41,64–66]. The bioactive compound identified to be the causal agent of photosensitivity in St. John’s Wort is the photocytotoxic and highly fluorescent pigment hypericin [32,64] (Figure 2). The leaves of this plant, which can reach two meters in height, are covered with small glands which contain this potent compound [31,64,67,68]. Threshold doses for onset of photosensitization in domestic livestock are suggested to be 3mg/kg dry matter (DM) in sheep [41] with a higher tolerance observed in cattle [69]. Hypericin photosensitization in domestic livestock manifests with classic clinical signs of UV photosensitivity including reddening and oedema of tissues of the muzzle, eyes and ears, along with increased rectal temperature [66].

Hypericin is a phenanthroperylenequinone which can form strong associations with a variety of proteins; its mode of action during photosensitization and potential as a photodynamic therapeutic have been well studied [31,70] (Figure 2). Photoactivated hypericin [71] is a potent generator of singlet oxygen (^1^O_2_), superoxide anions and other reactive oxygen species such as hydrogen peroxide (H_2_O_2_) [72–74]. All of these reactive oxygen species can induce cell death in UVA light-exposed tissues. Production of hypericin is stimulated by exposure to UVB and increased levels of total UV irradiation in greenhouse and field- grown plants [32]. It should be noted as well that exposure to light of longer wavelengths can result in generation of reactive molecules in plants and grazing livestock, and in some cases these longer wavelengths can penetrate tissues more effectively, thereby causing greater sensitivity and dermatitis [20]. Additional studies to investigate the impacts of light quality are warranted as very few studies assessing impact of various wavelengths of light have been performed, regardless of species.

Hypericin-generated reactive oxygen species give rise to cell loss in the dermis by apoptosis, necrosis and possibly by autophagy, but evidence for this is limited [75–79]. This process of cell damage relies on co-localization of the reactive oxygen species generated by hypericin and their cellular targets, as diffusion of hypericin-induced reactive oxygen species can occur only over short distances due to their unstable nature [75]. Hypericin has been shown to co-localize with the membranes of a variety of subcellular organelles including mitochondria, endoplasmic reticulum, Golgi apparatus, and lysosomes, suggesting that all of these critical intracellular structures may be the primary targets for this potent photocytotoxic compound [73,78,80–84]. As such, the presence of hypericin and light exposure results in degradation of SB cells of the dermis by reactive-oxygen species mediated cell death, causing the significant clinical photosensitization observed in affected livestock. Removal of animals from pastures containing St. John’s Wort is generally sufficient to reduce circulating hypericin levels and limit adverse photosensitization reactions in grazing animals [41,66].

### 5.2. Secondary Photosensitization: Hepatogenic Dysfunction in Grazing Herbivores

#### 5.2.1. The Role of the Chlorophyll Metabolite Phytoporphyrin in Hepatogenic Photosensitazion

Hepatogenic (secondary) photosensitization occurs when SPPs ingested by the animal cannot be removed efficiently from the systemic circulation due to disease or dysfunction of the liver [15]. A key player in secondary photosensitivity is the photoactive chlorophyll breakdown product phytoporphyrin, which builds up in significant concentrations in the bloodstream of affected grazing animals, thereby inducing photosensitivity (Figure 2). Phytoporphyrin is produced as a normal byproduct of digestive function in grazing livestock, when large quantities of chlorophyll consumed in the normal diet are broken down by bacteria in the gastrointestinal tract, generating significant levels of phytoporphyrin. Phytoporphyrin is absorbed from the stomach or rumen into the bloodstream where it is removed by the liver for conjugation and excretion in bile. Normal plasma concentrations in sheep have been shown to be less than 0.01 mM/L, with photosensitization occurring at concentrations >0.03 mM/L in affected animals [85]. If hepatic function is impaired, phytoporphyrin levels increase in the systemic circulation [36] and subsequently accumulate in the skin, where activation by sunlight results in damage to the sensitive dermal layers. Although the mode of action of phytoporphyrin is unknown, studies have shown binding of phytoporphyrin to Golgi apparatus and mitochondria, indicating that these organelles may be the primary target of this photocytotoxic compound [36,86].

The plant-derived toxins (excluding mycotoxins produced by parasitic or saprophytic fungi on plants) most often associated with secondary photosensitization belong to the following groups: steroidal saponins, terpenes and tannins. Other hepatotoxins may be associated with secondary photosensitization, but the association is less common, e.g., those plants containing PAs. Plant or microbial products associated with hepatogenic photosensitization include lantadenes, steroidal or lithogenmic saponins, mycotoxins (sporidesmin and phomopsin) and certain tannins [15]. These compounds cause photosensitization indirectly by damaging either hepatocytes and/or bile ducts, disrupting the liver’s ability to excrete phytoporphyrin into the gastrointestinal tract via the biliary system. This disruption results in hepatic injury, but injury is generally not severe enough to result in rapid death. Instead, affected herbivores usually exhibit some degree of cholestasis for at least a few days. There are a number of well recognized plant-derived hepatotoxins including steroidal saponins, terpenes and tannins that also can cause death via acute hepatic failure [15,17]; if these are ingested in smaller doses so that the animal survives the initial hepatic insult, ensuing hepatic changes may then result in secondary photosensitization. An example of this would be certain cases of plants in the *Myoporaceae* (Emu bush, turkey bush and boobialla) and associated poisoning [17]. Generally these plant toxins associated with secondary photosensitization cause subacute to chronic hepatic injury resulting in cholestasis. Other hepatotoxins may be associated with secondary photosensitization, but their presence is inconsistent or their structural elucidation remains unconfirmed.

#### 5.2.2. *Lantana* spp. and the Triterpenes

The genus *Lantana* (Verbenaceae) contains many species that are native to the Americas and Africa and has become naturalized as a noxious weed in tropical, subtropical, and warm temperate climates. *Lantana camara* L. is primarily associated with photosensitization (mostly the red, pink and orange varieties). *Lantana camara* consists of a species complex made up of highly variable hybrids that originated from a number of species originating in Mexico, Central America and the Caribbean Islands [17]. Lantana has now naturalized in nearly 50 countries, spreading over Australia, Asia, Africa, and the Americas and is recognized as a noxious species in many [87]. The plant produces a wide range of compounds including those classed as allelopathic. The most important of these allelopathic compounds are the triterpenes, which are thought to contribute to the predominance of lantana in various ecosystems and to loss of biodiversity [88]. This important chemical group is also primarily responsible for the severe jaundice and photosensitization seen within 1–2 days following consumption of *L. camara.* The triterpenes shown to be associated with hepatotoxicity in the field are lantadene A (primarily), lantadene C and icterogenin [17,88–90]. Another triterpene, the reduced form of lantadene A, is present in small amounts in *L. camara*, and was shown to be toxic after hepatic biotransformation, suggesting that the toxicity associated with the triterpenes may be associated with as yet unidentified biotransformed metabolites.

Although a large range of animals have shown to be affected experimentally, cattle are the most commonly affected species in the field, although sheep and horses have also shown photosensitization [91,92]. Ruminants typically show greater effects as plants retained in the rumen can exert toxic effects over a longer period of time than those processed by non-ruminants with only a simple stomach [17]. Neonatal lambs and calves appear resistant, and goats are unlikely to eat the plant [88]. Plants are consumed when there is limited choice (such as in times of drought) or when the animal is naïve; rumenal stasis occurs within hours of consumption via an inhibitory neural impulse from the damaged liver [93]. Experimentally, single large doses of the toxic principles by sheep can cause periacinar necrosis; however continuous exposure to lower doses of toxins is required for canalicular hepatocyte membrane damage and subsequent intrahepatic cholestasis [94,95]. This is facilitated by the initial rumenal stasis, with rumenal contents acting as a toxin depot for transport via the portal vein to the liver; significant photosensitization occurs as a direct result of liver cholestasis resulting from hepatic damage after ingestion of the plant [17].

#### 5.2.3. The Steroidal Saponins: Switchgrass (*Panicum* spp.) (Poaceae)

Switchgrass (*Panicum virgatum* L.) and other *Panicum* spp. have been associated with hepatogenous photosensitization in grazing herbivores in the USA and Australia. Recent studies by Lee *et al*. 2009 have shown that major saponins were noted in switchgrass and saponin concentration varied with switchgrass cultivar and plant part [94]. Highest levels were noted in actively growing leaf tissues. When studies were performed with switchgrass that was implicated in horse and sheep toxicity, several steroidal saponins were identified, including the primary sapogenin, diosgenin (Figure 2). Hepatogenous photosensitization has also been reported in a number of *Panicum* spp., including the invasive *Panicum effusum* R. Br. also known as *P. capillare*, in different regions around the world. In the western USA, photosensitization was associated with an abnormally hot and dry summer and exposure of grazing lambs for nearly a month [95]. Subsequent groups of lambs also experienced photosensitization within weeks of introduction to switchgrass pastures, with several showing dermal sensitivity and several mortalities out of a flock size of approximately 100 sheep. Mature ewes were unaffected, showing that maturity influenced toxicity and likely the ability to detoxify the associated saponins or exhibit greater dermal protection due to age and condition/pubescence of skin and coat. Interestingly, in subsequent years under normal temperatures and rainfall conditions, photosensitization was not observed. Careful monitoring of young animals grazing *Panicum* spp. is now suggested. In Australia, Merino sheep grazing on *P. capillare* or *P. colouratum* exhibited hepatogenous photosensitization. The condition is called “yellow bighead” and is characterized by swelling and drooping of ears, followed by swelling of the face, nose and lips, and eventually the whole head, accompanied by intense irritation, and tendency to seek shade. Eventually in the absence of shade the animal may develop jaundice and die [17,96].

#### 5.2.4. Photosensitization and Pyrrolizidine Alkaloids: Paterson’s Curse (*Echium plantagineum* L.) and *Senecio* spp

Pyrollizidine alkaloids (PA) may rarely give rise to hepatogenic photosensitization in livestock as they cause significant hepatic damage in animals ingesting plants containing high levels of these compounds [15]. They are included in this review to highlight the possibility of hepatogenous photosensitization secondary to toxic hepatic insult that does not directly affect the biliary excretory pathway (as the two preceding examples), but which may occasionally result in severe enough damage to the liver to have secondary effects on this pathway, and result in photosensitization. Pyrollizidine alkaloids are found in a wide variety of plant genera, with approximately 3% of all plant species known to contain certain pyrrolizidine alkaloids. More than 350 individual PAs have been identified to date [97–99]. Species particularly associated with hepatotoxicity in domestic livestock are in the *Echium*, *Senecio*, *Crotalaria* [15], *Cynoglossum*, *Amsinckia*, *Helioptopium* and *Trichodesma* genera [19]. These plants are generally in the Boraginaceae or Asteraceae families. PAs have been shown to have varying levels of hepatotoxic and genotoxic activity and, when ingested in sufficient quantity and over sufficient time, can cause severe acute and chronic liver dysfunction or failure, often resulting in death, as in the case of heliotrine produced by *Helitrope* spp. [38].

Pyrrolizidine alkaloids exhibit a common mode of action in affected grazing herbivores. They are metabolized by hepatocytes in the liver, particularly those of the centrolobular zone which have the highest enzymatic activity and are furthest from the vascular supply. These centrolobular cells contain high levels of enzymes catalyzing oxidation, reduction and hydrolysis reactions, which are associated with catabolism of toxins and generation of biologically active secondary metabolites [100]. PAs contain a pryrrolizidine nucleus which undergoes a dehydrogenation conversion in hepatocytes to create a pyrrole, the highly cytotoxic metabolite of PA (Figure 2) [101]. Pyrroles also exert their cytotoxic action by intercalating DNA and crosslinking proteins and amino acids eliciting both cytotoxic and anti-proliferative effects [98]. Pyrroles are highly active and, in general, exert their effects directly at the site of their generation, in the hepatocytes of the liver itself. However, not all pyrrole activity is restricted to the liver, and in the case of *Crotalaria* toxicosis, some circulation of PAs and their related catabolites has been suggested to be the cause of lung tissue damage often associated with this toxicosis [15,99].

Classic histopathological changes in the liver associated with PA hepatotoxicity include hepatocyte hypertrophy, necrosis, perportal necrosis, enlargement of the nucleus (megalocytosis) and fibrosis [15,102]. DNA crosslinking causes significant defects of cell proliferation; the hepatocytes of animals affected by PA toxicosis can be up to 30 times their normal size with evidence of significant DNA accumulation [15,98]. Hepatic dysfunction can result in death, but acute or chronic hepatic damage can result in photosensitization due to a failure of normal breakdown and excretion of phototoxic compounds via the liver.

##### 5.2.4.1. *Echium* spp

Paterson’s Curse (*Echium plantagineum*) (Boraginaceae) and other *Echium* spp. are known to cause hepatotoxic photosensitization [40]. There are marked differences in the sensitivity of domestic species to *Echium* toxicosis, with cattle and horses showing the highest levels of sensitivity and sheep exhibiting some tolerance to the compounds present in these plants [15,98,103]. Pyrrolizidine alkaloids such as echimidine (Figure 2) and echiumine are found in greatest quantity in *E. plantagineum*, among numerous other PAs which have been identified and quantified in stems, leaves and flowers [96,104]. These compounds are known to contribute to liver damage and photosensitivity in grazing livestock [3,96].

##### 5.2.4.2. *Senecio* spp

*Senecio* spp. (Asteraceae), the woody groundsels and hardy ragworts in particular, are a significant problem for livestock producers, being widespread in their distribution and highly toxic to ruminants and equines [15,40,98]. Unlike the PAs present in Paterson’s curse and other toxic species, the pyrrolizidine alkaloids found in *Senecio* spp. are not sensitive to microbial degradation in the rumen, so higher concentrations can pass into the circulatory system. One causal factor in this variable toxicity is the diversity in structure of the PA compounds present in these different plant species. One major bioactive PA present in *Senecio* is retronecine (Figure 2), which is a PA containing closed esters, as opposed to the open esters of echimidine and heliotrine associated with *Echium* spp. and *Heliotrope* spp. toxicosis, respectively. Another common PA in *Senecio* spp. is seneciorine; this compound is synthesized in the roots of living plants and translocated to the shoots as the plant develops. The closed ester PAs are much more resistant to enzymatic degradation by bacteria in the gut and result in increased enzymatic conversion of pyrroles in hepatocytes with species dependent differences [15,40]. As with *Echium* spp., their cytotoxic effects in the liver increases circulating levels of phytoporphyrin, causing dermal photosensitivity in animals ingesting sub-clinical or acute toxic levels, even though these compounds do not exhibit a direct effect on dermal tissues of grazing herbivores.

### 5.3. Biserrula (*Biserrula pelecinus*)—A Novel Photosensitizing Plant

*Biserrula pelecinus* is an annual pasture legume native to the southern Mediterranean. It was first introduced to Australia in 1991 as a potentially valuable rotational pasture species for livestock production, particularly in low rainfall zones [105–107]. It produces large quantities of biomass, exhibits drought tolerance and is effective for weed suppression in pasture rotations. However, despite proving to be a valuable addition to the pasture toolbox, producers in Australia have reported incidence of severe photosensitization in sheep grazing on *Biserrula* pastures. *Biserrula* photosensitization, anecdotally, appears to be associated with a particular phase of crop maturity (late flowering) and shows an increased severity of clinical signs in naive or young animals grazing pastures predominating in biserrula. Two varieties of biserrula are currently in use in Australia, “*Mauro*” and “*Casbah*” [108,109]; previous reports and our own studies have shown both varieties cause photosensitization in sheep (Figure 4) [110]. The etiology of this photosensitization and the causal compound(s) are, as yet, unknown, however, rapidity in onset of symptoms and absence of significant liver pathology in affected animals suggests a primary photosensitizing agent may be responsible. Studies are ongoing to identify this agent and to further characterize the pathogenesis of photosensitization caused by this recently introduced species.

## 6. Conclusions

The impacts of photosensitizing SPPs can lead to sporadic and/or significant breakouts of photosensitization in grazing herbivores, with some leading to considerable economic losses for livestock producers. The specific impact of environment and genotype on production of these light- absorbing bioactive molecules in plants is still not well understood, but they are produced in both crops and invasive weeds resulting in photosensitization to grazing herbivores. Photosensitivity occurs in animal cells as series of reactions that are mediated by a light- absorbing molecule, specifically in this case plant-produced metabolites that are commonly heterocyclic or polyphenolic. In sensitive animals, associated photoreactions are generally most severe in non-pigmented skin which has the least protection from light exposure. Other molecules cause serious damage to the liver, resulting in inability to remove metabolites of chlorophyll from the blood stream in affected animals. Recent advances in our ability to identify and detect SPPs at trace levels, both in the plant and surrounding environment or in organisms that ingest the plant, have provided new insights as to the roles of secondary plant metabolites in photosensitization in grazing herbivores. As we improve our ability to detect and characterize SPPs that cause photosensitivity in trace quantities, we can more effectively determine the specific modes of action of these compounds. Future research in this area is warranted in order to successfully manage grazing pasture and rangelands more effectively, and develop a fundamental understanding of the factors impacting production of photosensitizing SPPs in higher plants and their effects on the animals that graze them.
